# The synaptic organization of the human temporal lobe neocortex by high-resolution transmission, focused ion beam scanning, and electron microscopic tomography

**DOI:** 10.1007/s12565-025-00900-y

**Published:** 2025-09-23

**Authors:** Astrid Rollenhagen, Joachim H. R. Lübke

**Affiliations:** 1 Institute of Neuroscience and Medicine INM-10, Research Centre Jülich GmbH, Leo-Brandt Str, 52425 Jülich, Germany; 2 Department of Psychiatry, Psychotherapy and Psychosomatics, Medical Faculty, RWTH University Hospital Aachen, Pauwelsstr. 30, 52074 Aachen, Germany; 3 JARA Translational Brain Medicine, Jülich-Aachen, Germany

**Keywords:** Human temporal lobe neocortex, Quantitative three-dimensional models of synaptic boutons, Synaptic vesicles, Transmission and focused ion beam scanning EM, EM tomography

## Abstract

Fine-scale transmission electron microscopy (TEM), focused ion beam scanning EM (FIB), and EM tomography have opened a new window on the synaptic organization of the normal, developing and pathologically altered brain in experimental animals. Progress in the human brain has been slower, due to technical challenges and the problem of tissue availability from donors that underwent epilepsy or tumor surgery. The present manuscript is in part an overview of the geometry of synaptic boutons in surgical biopsy samples taken from human temporal lobe neocortex (‘hTLN’). Here, the number, size, and shape of active zones (the equivalent of functional neurotransmitter release sites) and the three functionally defined pools of synaptic vesicles were quantified, with comparisons to the same parameters in experimental animals. High-resolution TEM tomography further allowed new insights concerning the readily releasable pool of synaptic vesicles, one of the key structural elements in synaptic transmission and plasticity. The quantitative 3D models of synaptic boutons provide the basis for numerical and/or Monte Carlo simulations of various signal cascades underlying synaptic transmission that at least in humans are still only partially accessible for experiment. In a second focus, we provide a step-by-step walk-through with illustrations of basic methodology for tissue preparation and analysis, for both TEM and FIB-SEM, including a thorough discussion of the main advantages and disadvantages of the several techniques and the particular challenge of working with human tissue.

## Introduction

For centuries, the belief that the structure of the brain and its elements reflect its function has been a ‘driving force’ for investigations and the source of major discoveries in neuroscience, leading to thousands of publications and numerous textbooks.

Synaptic boutons (SBs) are fascinating structures that govern and modulate the computational properties of any given network in the brain. They can act as amplifiers, discriminators, and integrators, can rapidly switch their mode of release upon different up- and down-state conditions, and are intricately involved in the sensory perception of our body and the surrounding world. How this is reflected in their structural composition still remains in part unresolved in quantitative details, in particular in humans.

The motivation to work in the human neocortex is threefold: First, the ultimate goal was to describe the synaptic organization of the cortical column, the smallest function unit of the neocortex (Hubel and Wiesel 1963, 1969; reviewed by Rakic 2008; Helmstaedter et al. 2009; Rockland and DeFelipe 2018) layer-by-layer, initially using healthy tissue and, later, based on our previous findings the pathologically altered human temporal neocortex (TLN) as a model system (see for example Yakoubi et al. 2019a, b; Cano-Astorga et al. 2021, 2023, 2024; Schmuhl-Giesen et al. 2022; Shapson-Coe et al. 2024; Rollenhagen et al. 2025). Second, to date, still only a few comprehensive and coherent quantitative morphological studies of SBs in the brain exist, even in experimental animals and different species (see for example Xu-Friedman et al. 2001; Marrone et al. 2005; Xu-Friemann and Regehr 2003; Rollenhagen et al. 2007, 2015, 2018; Dufour et al. 2016; Bopp et al. 2017; Hsu et al 2017; Prume et al. 2020). Third, in contrast to experimental animals, comparably little is known about the synaptic organization of the human brain, in particular about the quantitative morphology of SBs. Finally, our studies provide the basis to compare ‘normal’ non-affected human brain tissue with tissue taken from epileptic or tumor patients. Thus, these investigations represent a fundamental step to better understand synaptic complexes embedded in different brain networks in health and disease, particularly in the human brain.

High-resolution transmission electron microscopy (TEM) is one option to investigate the brain at the most fine-scale EM level and has thus opened a new window on the structural and synaptic organization of the brain. Further developments in high-end fine-scale TEM (see for example Rollenhagen et al. 2007, 2015, 2018, 2020, 2025; Bopp et al. 2017; Hsu et al. 2017; Rodriguez-Moreno et al. 2018; Yakoubi et al. 2019a, b; Schmuhl-Giesen et al. 2022), focused ion beam scanning electron microscopy (FIB-SEM: Alonso-Nanclares et al. 2008; Rollenhagen et al. 2020; Cano-Astorga et al. 2023, 2024; Shapson-Coe et al. 2024) and TEM tomography (Yakoubi et al. 2019a, b; Schmuhl-Giesen et al. 2022; Rollenhagen et al. 2020, 2025) extended these investigations further to the sub-cellular and even molecular level in the human brain. However, such detailed and quantitative investigations of SBs in humans became possible only by the availability of donated human biopsy tissue samples taken from epilepsy and tumor surgery. By contrast, so-called ‘post-mortem’ brains are only partially suitable for investigations at the cellular and sub-cellular EM level due to the comparably ‘poorer’ ultrastructural preservation of tissue samples obtained from post-mortem brains. This can be mainly attributed to hypoxia-mediated autolysis of biological membranes within the long time-window between the decease of the patient and the removal of the brain by the pathologist. This limits the use of such tissue for the generation of detailed quantitative 3D models of synaptic structures as described here (but see Cano-Astorga et al. 2021, 2023, 2024).

The human temporal lobe neocortex (‘hTLN’) is ideally suited for detailed structural investigations of the human brain, since it has to be removed to access the hippocampus proper during epilepsy surgery. Furthermore, the ‘hTLN’ occupying about ~ 20% of the total volume of the human cerebral cortex is of importance, because it represents a highly specialized associative, homotypic granular, six-layered neocortex involved in auditory, visual, vestibular, linguistic, and olfactory processing that is linked to other multimodal associations areas like the limbic cortex and various other sensory systems. Finally, the growing interest in working on the ‘hTLN’ is based on its involvement in several neurological diseases, most importantly as the area of origin and onset for the most common form of epilepsy, the temporal lobe epilepsy (Allone et al. 2017, reviewed by Tai et al. 2018). Thus, the TLN represents an important brain region in the normal and pathologically altered human brain.

One major and important question in synaptic neuroscience is whether findings about the structural compositions of the developing and pathologically altered brain of adult experimental animals can be transferred one-to-one to the human brain. Hence, the aim of the present review is to demonstrate that high-end, high-resolution TEM, FIB-SEM, and TEM tomography are useful tools to investigate the synaptic organization of the human brain. Here, we demonstrate that brain tissue samples taken and fixed immediately after excision during epilepsy surgery are structurally extremely well preserved. This lead to reliable and reproducible results in the investigation and quantification of the neuronal and synaptic organization of the normal and pathologically altered human neocortex.

## Protocols

### Materials and methods

#### Human neocortical tissue processing for TEM, FIB-SEM, and EM tomography

Biopsy tissue samples were obtained from both male and female patients (25–63 years in age) who suffered from drug-resistant temporal lobe epilepsy and underwent surgery to control their seizures. The consent of the patients was obtained by written statements and all experimental procedures were approved by the ethical committees of the Rheinische Friedrich-Wilhelms-University/University Hospital Bonn (ethic vote of the Medical Faculty to Prof. Dr. med. Johannes Schramm and Prof. Dr. rer. nat. Joachim Lübke, Nr. 146/11), and the University of Bochum (ethic vote of the Medical Faculty to Prof. Dr. Med. Marec von Lehe and Prof. Dr. rer. nat. Joachim Lübke, Reg. No. 5190–14-15; ethic vote of the Medical Faculty to Dr. med. Dorothea Miller and Prof. Dr. rer. nat. Joachim Lübke, Reg. No. 17–6199-BR), and the EU directive (2015/565/EC and 2015/566/EC) concerning working with human tissue.

**Fig. 1:**
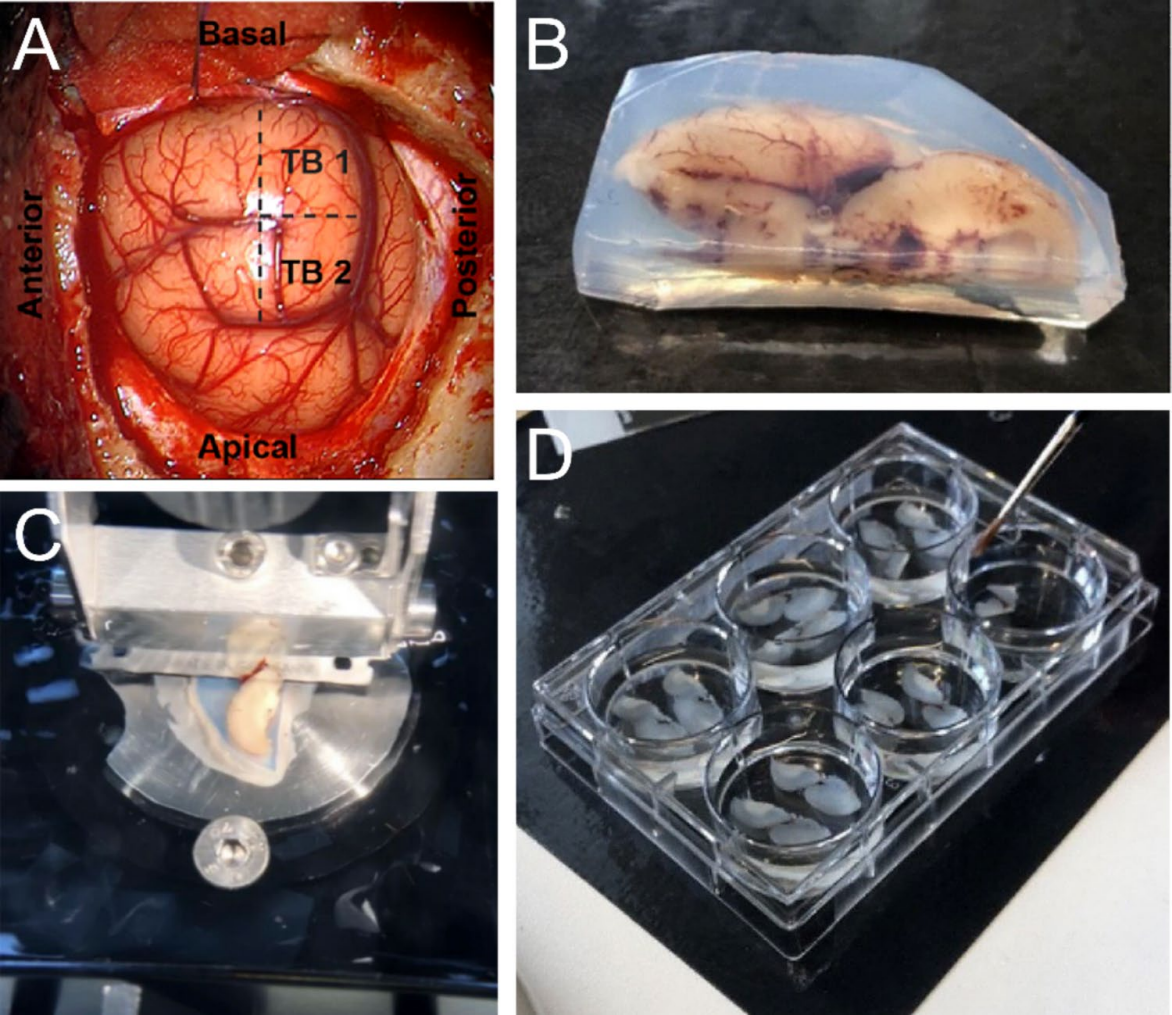
Removal, embedding, vibratome sectioning, and collection of the human tissue samples. **A,** View through the surgical microscope of part of the temporal lobe neocortex (gyrus inferior and medialis) during epilepsy surgery. T1 and T2 are the neocortical areas that were removed for EM embedding and analysis; **B,** prior to vibratome sectioning neocortical tissue samples were embedded in 5% Agar–Agar; **C,** vibratome sectioning of the neocortical tissue block in the frontal plane; **D,** collecting the vibratome sections in 6-well plates in PB (with permission from Springer Protocols. Taken from New aspects in analyzing the synaptic organization of the brain. Chapter 12: Quantitative Analysis of the Synaptic Organization of the Human Temporal Lobe Neocortex by Astrid Rollenhagen, Kurt Sätzler and Joachim H.R. Lübke)

During epilepsy surgery, blocks of neocortical access tissue from different regions of the superior, medial, and inferior temporal gyrus were taken far from the epileptic focus and may thus be regarded as non-affected (non-epileptic) tissue as routinely monitored by preoperative multielectrode electrophysiology and magnetic resonance imaging. In addition, functional studies using the same experimental approach (Mohan et al. 2015; Molnár et al. 2016; Seeman et al. 2018a, b; Obermayer et al. 2018; reviewed by Mansvelder et al. 2019) have clearly demonstrated that neocortical access tissue samples taken from epilepsy surgery do not differ in electrophysiological properties and synaptic physiology when compared with acute slice preparations in experimental animals. Non-epileptic neocortical access tissue, epileptic tissue (Fig. 1A), and the underlying hippocampus (not shown) were removed by the neurosurgeon listed above. Directly after their removal tissue blocks (average size ~ 5–7 × 5–7 cm) were incubated in ice-cold physiological saline and then further trimmed down to 1.5–2 cm coronal sections which were then either transferred to ice-cold appropriate fixatives (see below) or to sterile double-distilled water for Golgi-impregnation. The fixation procedure was done by Prof. Joachim Lübke who was always present at the operations. The time between the removal of the biopsies from the brain and fixation does not exceed 3–4 min to prevent hypoxia of the tissue samples and to guarantee a good ultrastructural preservation.

#### Fixation of biopsy tissue samples

After their removal from the brain, biopsy tissue samples of the gyrus temporalis of the TLN were immediately immersion-fixed in ice-cold, phosphate-buffered or cacodylate-buffered (0.1 M PB or 0.15 M CB pH 7.4) 4% paraformaldehyde and 2.5% glutaraldehyde at 4 °C. After 4 h the fixative was replaced by the same but freshly prepared solution and stored for 24–72 h at 4 °C. The fixation time should not exceed 72 h to prevent over-fixation. Hence, sections were then transferred to either PB or CB and stored at 4 ℃ until further use.

#### Vibratome sectioning of the tissue samples

After several washing steps (3–5 × 10 min each) in PB or CB tissue samples were then embedded in 4% agar–agar dissolved in PB or CB (Fig. 1B) and after hardening trimmed with a double-edge razor blade to remove excess of agar–agar. This embedding was done to stabilize the tissue block during sectioning. Then, vibratome sections (150–200 µm in thickness, VT1000S, Leica Microsystems GmbH, Wetzlar, Germany) were cut in the frontal (coronal) plane through the ‘hTLN’ with a double-edge razor blade (Fig. 1C) and collected and transferred with a paintbrush either to ice-cold PB (for TEM analyses) or to 0.15 M CB + 2 mM CaCl_2_ (for FIB-SEM analyses) in 6-well plates (Fig. 1D). Subsequently, two different protocols were used to prepare the tissue samples for either TEM or FIB-SEM.

### Processing for TEM

After thorough washing in PB, (3 × 10 min) sections were post-fixed for 30–60 min in 0.5% or 1% osmium tetroxide (Sigma, Munich, Germany) diluted in PB-buffered sucrose (300 mOsm, pH 7.4) at room temperature in the dark. After visual inspection under a light microscope and thorough washing in PB, sections were covered with round coverslips in multi-well plates to prevent distortions of the sections caused by the dehydration process. They were then dehydrated in a series of ethanol starting at 20–90% ethanol (15 min for each step), followed by 20 min in 95% ethanol and absolute ethanol (2 × 30 min). This was followed by a brief incubation in propylene oxide (twice 2 min; Fluka, Neu-Ulm, Germany). Sections were then transferred into a mixture of propylene oxide and Durcupan™ resin (2:1, 1:1 for 1 h each; Fluka, Neu- Ulm, Germany) and stored overnight in pure resin (mixture according to the manufactures’ protocol). The final pure Durcupan H™ solution was prepared from 100 g of component A, 100 g of component B, 3 g of component C, and 3 g of component D. The next day, they were flat-embedded either on coated glass slides or between Acla™ foils in fresh Durcupan™, coverslipped, and polymerized at 60 °C for 2 days. As already mentioned above the coverage of vibratome sections with thin coverslips, depending on the well diameter of the multiwall plates, it is important to keep the section absolutely flat, since folds can have an important impact on the semi- and ultrathin cutting process due to the unevenness in the tissue block. Another critical step is the transfer of sections from absolute ethanol to propylene oxide (preventing drying out of sections) and the time within propylene oxide to prevent too much hardening, which can cause difficulties during semi- and ultrathin sectioning. Finally, the transfer from the relatively large vibratome sections from the glass flasks to either the Acla™ foils or silicon-coated glass slides could be problematic because the hardening process makes the sections very fragile, and hence, they could break. Remedy: heat up the resin to become more fluid and use a fine brush or a tooth stick to transfer the sections from the glass flask to the Acla™ foil or silicon-coated glass slide.

### Processing for FIB-SEM

Tissue samples were fixed and cut as already described above. Vibratome sections were collected in 0.15 M CB + 2 mM CaCl_2_ in multi-well plates and thoroughly washed several times in the same buffer solution (5 × 3 min). Thereafter, sections were incubated in 1.5% potassium ferricyanide, 2% osmium tetroxide, and 2 mM CaCl_2_ diluted in CB for 1 h on ice. This was followed by a treatment in 1% aqueous thiocarbohydrazide for 20 min at room temperature (RT) and another subsequent incubation for 30 min in 2% aqueous osmium tetroxide solution at RT. Then sections were block contrasted with filtered aqueous 1% uranyl acetate overnight at 4 °C before they were contrasted with Walton’s lead aspartate staining (aqueous solution of 20 mM lead nitrate in 30 mM aspartic acid) for 30 min at 60 °C. In between all incubation steps, sections were thoroughly washed with the pure next diluent. After washing with deionized H_2_O, (5 × 3 min) section was dehydrated through an ascending series of ethanol dilutions on ice (30%, 50%, 70%, 90%, 100%; 5 min for each step, followed by 2 × 10 min 100% ethanol dried on a molecular sieve). Next, sections were transferred to pure propylene oxide (PO; 2 × 10 min) and then processed through a series of Durcupan H™: PO mixtures with ascending resin concentrations (1:4, 1:2, 4:1; 2 h, each and pure resin overnight, followed by again pure resin for 2 h). The final pure Durcupan H™ solution was prepared as described above. After resin infiltration, sections were flat-embedded either between Acla™ foils or between a silicon-coated glass slide and an appropriate piece of overhead transparency before samples were polymerized at 60 °C for 2 days. Critical steps for the tissue processing are the same as described above.

### Semithin sectioning for TEM

In the polymerized neocortical tissue samples, a region of interest (ROI) was selected by visual inspections under × 4 to × 20 magnification with a light microscope (Olympus BX10; Germany) and glued with a tissue adhesive on a pre-polymerized resin block. After further trimming with a razor blade to the final size of a triangulated rectangle, 0.5–1 µm semithin sections were cut with a Leica Ultracut S ultramicrotome (Leica Microsystems, Vienna, Austria). After toluidine-blue (1% in distilled water 30 s to 1 min) counterstaining, semithin sections were carefully washed in distilled water, dried on a hotplate and inspected under a light microscope at × 4 to × 40 magnification to check for the quality of structural preservation assessed by the intactness of neuronal somata, astrocytes, and other structures, and the absence of large holes within the neuropil. The final ROI was selected for serial ultrathin sectioning (55 ± 5 nm in thickness, silver to light gray interference contrast appearance) and sections were collected on Formvar™-coated slot copper grids (Plano, Wiesbaden, Germany).

### Protocol for Formvar™-coated slot copper grids

For TEM examination and TEM tomography Formvar-coated slot copper grids were used. First, glass slides were cleaned in absolute ethanol and sprayed dust-free using a dust cleaning agent. Then, the Formvar™ solution (0.25–3% diluted in either dichlorethane or dichloroformthane; Plano, Wiesbaden, Germany) was filled into a staining cuvette and the cleaned glass slide was dipped into the cuvette (approx. 2 s) and was then pulled out quickly and evenly because the thickness of the Formvar film critically depends on this procedure. Afterward, the glass slide was left to dry vertically and dust-free for about 10 min. Then, a large round beaker was filled with double-distilled water up to the rim. The water surface was cleaned with a glass rod from dust or other particles. To avoid sticking of the Formvar film to the glass slide a razor blade run carefully along the lower edges of the glass slide. Then, breathe on the filmed glass slide and, while the surface is still moist, slide the narrow side of the glass slide into the water at an angle of approx. 25° until the Formvar film from both sites of the glass slide is completely detached swimming on the water surface. The thickness and quality of the Formvar film should be checked using their interference color, only Formvar films that appear silver to gray and without holes should be used. The best time for producing Formvar-coated slot copper grids is on a dry and humid day. Using fine tweezers, slot copper grids with their shiny side up, were then placed on top of Formvar^−^film floating on the water surface. When the surface of the Formvar film is covered entirely with slot copper grids, the foil is covered with a slightly larger Parafilm while rolling it gently on the water surface to pick the film up. Then, the strips of Parafilm™ covered with the Formvar film and the underlying slide meshes were transferred to Petri dishes to let them air-dry and to keep them dust-free until further use. Finally, pour the Formvar^−^solution from the cuvette back into a light-protected storage bottle at room temperature.

### Serial ultrathin sectioning for TEM

For the generation of quantitative 3D models of SBs and their target structures, individual series were comprised of ~ 70–170 ultrathin sections to allow the reconstruction of SBs of different sizes terminating on individual dendritic segments or spines. SBs were judged to be complete if the axon gave rise to an end terminal SB, or if the axon could be followed in both directions with a swelling leading to an *en passant* SB which can be unequivocally identified from the beginning to its end in a series of ultrathin sections. Prior to TEM examination, sections were processed either with uranyl acetate or UranyLess EM™ (Sciences Services, Munich, Germany; 15–20 min) and stained subsequently with lead citrate (3–5 min) using a standard protocol by Reynolds (1963). Serial ultrathin sectioning requires a highly skilled technician with long-time experience in serial ultrathin sectioning. The main problem is not to lose sections within a series and to keep the order of sectioning. Additionally, only intact Formvar films without large holes should be used to avoid distortion and tearing of the film during TEM exposure.

### TEM data acquisition

**Fig. 2:**
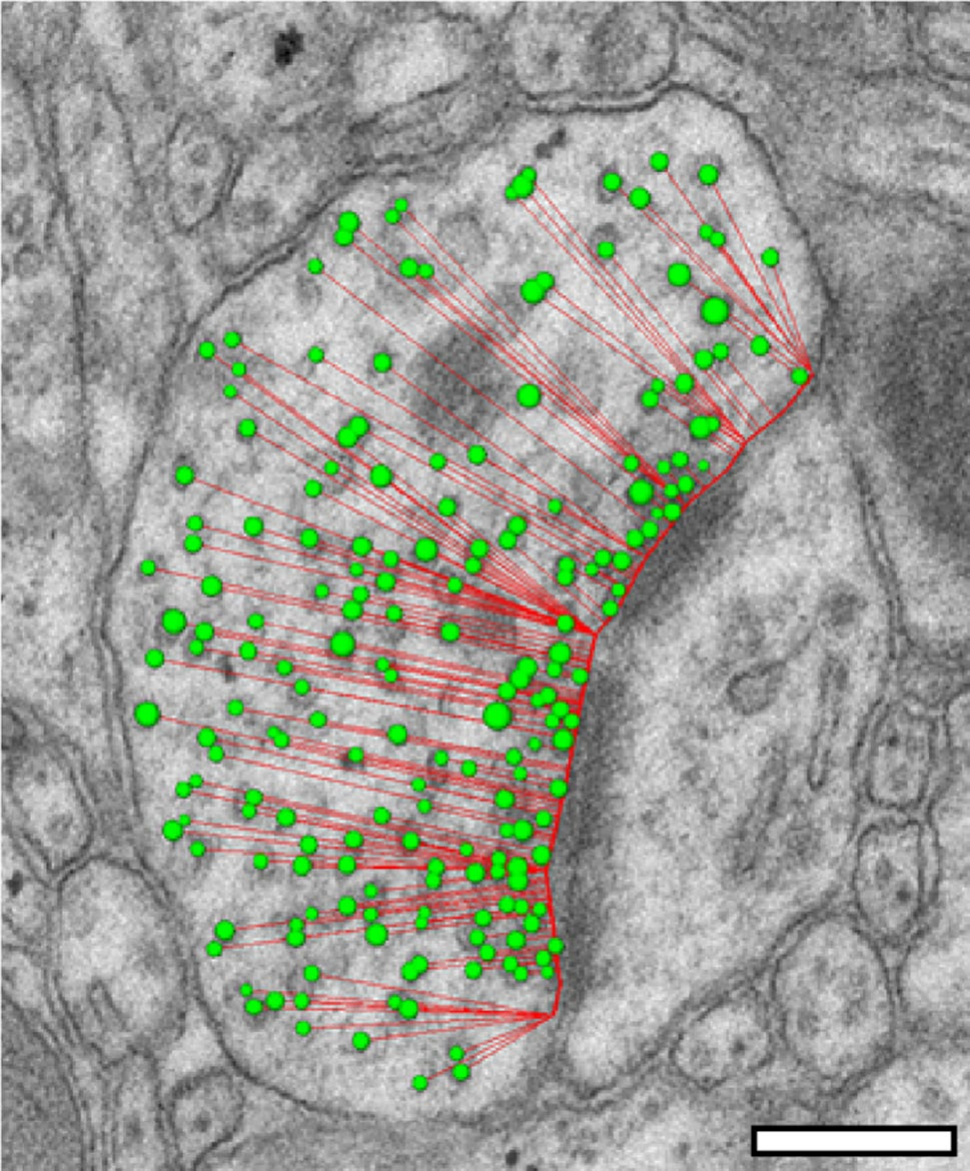
SV distance analysis. In individual SBs, the diameter of each SV was measured by an algorithm-driven software tool implemented in Open-CAR. Scale bar 0.25 µm. Subsequently, in a modified Scholl analysis, SVs were divided into the following perimeter-categories (p) at 10 nm intervals: putative RRP p10 nm, putative RRP p20 nm, putative RP (p60–200 nm), and putative resting pool *p* > 200 nm. Scale bar 0,25 µm

**Fig. 3:**
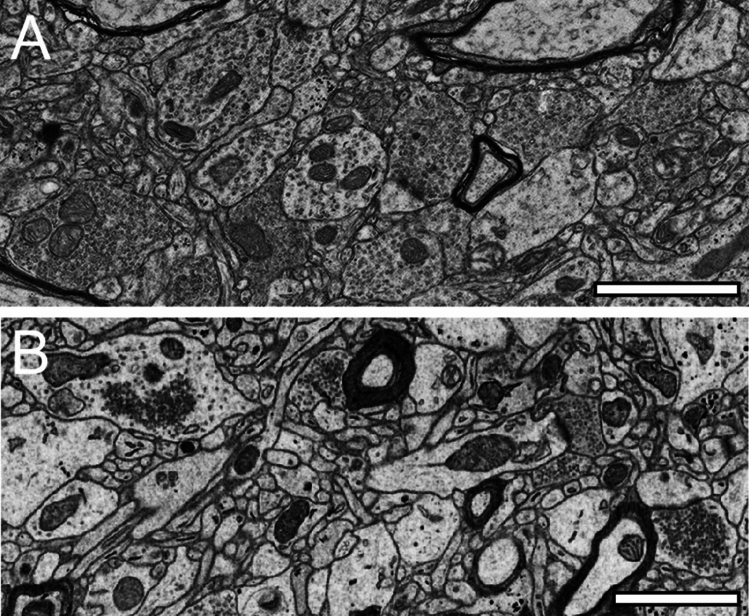
Synaptic organization of the neuropil in the ‘hTLN’. **A,** Low-power EM micrograph of the neuropil of L2 in the gyrus temporalis medialis of the ‘hTLN’ processed for TEM. Within the neuropil, numerous dendritic profiles and SBs of different shapes and sizes, identifiable by their SV content, can be identified. Some SBs form synaptic complexes with dendritic shafts or spines. **B,** Low-power EM micrograph of the neuropil, similar in its organization as in (**A**), of L5 in the gyrus temporalis medialis of the ‘hTLN’ processed for FIB-SEM. Note the different structural appearance of the neuropil, in particular those of AZs and the pool of SVs between the TEM (**A**) and FIB-SEM (**B**) processed tissue samples. Scale bar in A and B, 1 µm

Synaptic boutons and their target structures neurons were photographed from a series of ultrathin sections at a primary magnification of 8000x (Zeiss Libra 120, Oberkochen, Germany) using a Proscan 2 K digital camera and the Panorama Function of the SIS Analysis (Olympus Soft Imaging System, Münster, Germany) or Image SP (Fa. Tröndle, Mohrenweis, Germany) software. Data were stored in a database until further use. In addition, interesting details about the synaptic organization within different layers of the neocortical gray matter and gyri were photographed at different magnifications for further documentation within figure illustrations (see Figs. 2A, 3, 5). Care was taken not to select more than 2–3 ROIs in individual series in order not to stress the Formvar film and individual sections too much during high-voltage TEM illumination and also to increase the chance of a second TEM examination.

### TEM tomography

For TEM tomography, 200–300 nm-thick sections were cut from blocks prepared for ultrathin sectioning generated according to the above-stated protocol. These sections were also collected on Formvar-coated but line grids (Plano, Wiesbaden, Germany) to reach the maximum stability during the illumination and data acquisition process.

TEM tomography was performed to quantify synaptic vesicles (SVs) particularly belonging to the readily releasable pool (RRP). Sections were mounted on Formvar-coated line grids and were counterstained with uranyl acetate and lead citrate following a slightly modified staining protocol by Reynolds (1963). Subsequently, sections were examined with a JEOL JEM 1400Plus, operating at 120 kV and equipped with a 4096 × 4096 pixels CMOS camera (TemCam-F416, TVIPS, Gauting, Germany). Tilt series were acquired automatically over an angular range of − 60° to + 60° at 1° increments using Serial EM; Ver. 3.58. Stack alignment and reconstruction by filtered back projection were carried out using the software package iMOD, Ver. 4.9.7. During the alignment procedure, the stack was binned in x–y dimension by 2 pixels to reduce the noise and to increase the contrast of the final reconstruction.

### FIB-SEM data acquisition

Based on the initial macroscopic appearance under a light microscope (equipped with × 10 to × 40 objectives) of the vibratome sections, ROIs were cut out of the resin-embedded tissue sample using a 4 mm biopsy puncher or a razor blade before they were glued with superglue onto a pre-polymerized resin block, using freshly prepared Durcupan™. After overnight polymerization at 60 °C, the mounted sample was trimmed precisely to the ROI. An excess of resin on top of the tissue was removed using a histology diamond knife on an ultramicrotome (UC7, Leica Microsystems, Vienna, Austria) leading to a smoothly polished surface providing an optimal basis for the following FIB-SEM preparation steps. Next, the trimmed sample was removed from the underlying resin block with a razor blade and then glued onto an SEM aluminum specimen stub using a two-component conductive silver epoxy (Silver Conductive Epoxy, Ted Pella, Redding, USA). The epoxy was cured at room temperature overnight, sputter-coated with a platinum/palladium layer of 20 nm thickness and finally transferred into the FIB-SEM (Crossbeam 540, Carl Zeiss, Oberkochen, Germany) for 3D analysis.

A coarse trench was milled with the FIB at 30 kV/65 nA, polishing of the surface was performed at 30 kV/15 nA, and fine milling for data acquisition was performed at 30 kV/7 nA. The cross-section surface was imaged with an electron energy of 2 keV and an electron beam current of 500 pA (“analytical setting” of the column electron optics) using an in-column energy-selective backscatter electron detector. The dwell time was 10 ms with line average 1. The pixel size in the x–y-plane was 5 nm and the slice thickness (z-direction) was 50 nm yielding a voxel size of 5 nm × 5 nm × 50 nm. The image acquisition software Atlas 3D (Ver. 5.2.0.125, ZEISS, Oberkochen, Germany) allowed the automated collection of 3D-SEM datasets using automated correction algorithms for drift, focus, and astigmatism. A final alignment of the resulting image stack was carried out to the serial section alignment workflow of the software package iMOD, Ver. 4.9.7.

For a direct comparison between the TEM and SEM images, single sections of the FIB-SEM run were acquired using high-resolution SEM parameters, in detail, an electron energy of 1.5 keV at a beam current of 111 pA in ‘high resolution’ setting of the column electron optics. The pixel sizes and dwell times were chosen to achieve the best resolution and signal-to-noise ratio in the resulting image. Setting the dimension of the ROI not to exceed 50 × 50 µm is critical to sufficient digital image quality and the milling process.

### 3D-volume reconstructions of SBs

Digital images acquired with either TEM or FIB-SEM were imported into OpenCAR, stacked and transformed linearly, such that corresponding structures were aligned along all consecutive images comprising the 3D image stack. In such individual z-stacks, all structures of interest, SBs and their target structures, were defined by different colors and codes. After contouring throughout all images, 3D-volumetric reconstructions were performed from which surface and volume measurements were obtained.

To generate quantitative 3D models of SBs and their target structures all calculations were performed offline using an open-source batch version of OpenCAR, which generates 3D-volume reconstructions as well as space-delimited tables for each measurement that are readable by standard analysis software. For further details on 3D-volume reconstructions, see Sätzler et al. (2002) and Rollenhagen et al. (2025).

In addition to the surface and volume of SBs, the surface areas of the pre- (PreAZ) and postsynaptic density (PSD) were measured. Per definition, the PreAZ and PSD constituting the AZ are regions of densely, electron-dense, dark-appearing material, condensed at the pre- and postsynaptic apposition zone. The surface areas of the PreAZ and PSD were computed separately by first generating a 3D surface model of the SB. The PreAZ was then measured by extracting this area from the reconstructed presynaptic bouton membrane that was covered by this membrane specialization (i.e., where the contour line coincided with less than 30 nm distance from the presynaptic membrane). Hence, the length (l) of the PreAZ (l PreAZ) and the surface area (SA) of the PreAZ (SA PreAZ) is already known. The size of the PSD opposing the PreAZ was estimated under the following assumptions: 1) both membrane specializations, PreAZ and PSD, run parallel to each other at the pre- and postsynaptic apposition zone; 2) for both membrane specializations a contour line was drawn determining their actual length (l PreAZ and l PSD). Hence, the surface area of the PSD (SA PSD) is estimated by the following equation:

$$SA \, PSD \, = \, SA \, \Pr e \, * \, l \, PSD \, / \, l \, \Pr eAZ,$$

which is the perimeter ratio between the outlines of the PSD to that of the synaptic contact.

The synaptic cleft width was measured at the two lateral edges and the center of the PreAZ and PSD on digital electron microscopic images or directly during EM inspection using the measurement tool software of ImageSP (Fa. Tröndle, Mohrenweis, Germany). Only synapses in which the AZ was perpendicularly cut, and which showed the typical broadening of the synaptic cleft were included in the sample. The two values for the lateral edges were averaged and a mean ± SD was calculated for each synaptic cleft. Finally, a total mean ± SD over all individuals was given.

### Analyzing vesicle distribution and pool sizes

Synaptic vesicles (SVs) play an important role in synaptic transmission. To obtain estimates for their size and distance distribution, each SV in an SB was marked by a line, thereby measuring its diameter and the minimal distance between its outer membrane and the PreAZ using an algorithm implemented in OpenCAR (Fig. 2). This distance gave an estimate of the minimal 2D distance that a vesicle had to bridge before it ‘touches’ the membrane specialization. As the minimal 2D distance for each vesicle to the PreAZ was measured, it was possible to define different pools of SVs located at individual PreAZs (Fig. 2). To define morphological correlates for the three functionally defined pools of SVs, in a modified Scholl analysis, SVs were divided into categories at 10 nm intervals (perimeter [p]). Using established criteria (reviewed by Rizzoli and Betz 2005; Denker and Rizzoli 2010), the different pools were sorted as follows: p10 nm putative RRP, p20 nm putative RRP, p60–200 nm putative RP, *p* > 200 nm putative resting pool. Errors in estimates of SV numbers were not applied for the numbers of small clear vesicles reported in this study. For large dense-core vesicles, double counts were excluded by careful examination of adjacent images and were only marked in the image where they appear largest.

### Statistical analysis

From the numerous 3D-volume reconstructions and spreadsheets computed by OpenCAR, statistical summaries and graphs were generated automatically using special purpose functions written for the freely available statistics package R (R Development Core Team. R: A language and environment for statistical computing. R Foundation for Statistical Computing, Vienna, Austria. ISBN 3–900,051-07–0, URL http://www.R-project.org, 2005). Additionally, the non-parametric Kruskal–Wallis H-test analysis with post hoc U tests was used (GraphPad InStat Software Inc., San Diego, CA, USA or PAST 4.02 [Hammer et al. 2001]) to obtain differences between several structural parameters investigated.

## Results

Our main and ultimate goal is to establish the quantitative synaptic organization of a cortical column, layer by layer, exemplified for the ‘hTLN’ in the normal (Yakoubi et al. 2019a, b; Schmuhl-Giesen et al. 2022; Rollenhagen et al. 2020, 2025) and pathologically altered brain (work in progress). The present review will give a more general view onto the synaptic organization of the ‘hTLN’ but demonstrates the possibility to generate quantitative 3D models of SBs and their target structure using both TEM and FIB-SEM data sets.

Research on the human brain was for a long-time restricted to so-called ‘post-mortem brains’ via donations after the decease of the patient. However, it turned out that this tissue is only partially suitable for fine-scale, high-resolution TEM and FIB-SEM due to the comparably ‘poorer’ ultrastructural preservation of biological membranes caused by the ongoing autolysis of the tissue. Even if the time between the decease of the donor and the removal of the brain was within 3–4 h of death with immediate post-fixation, not all synaptic parameters can be unequivocally determined (see Domínguez-Álvaro et al. 2019, 2021, 2023, 2024; Cano-Astorga 2021; Shapson-Coe et al. 2024). To overcome this problem, non-epileptic access tissue from epilepsy or brain tumor surgery became an excellent alternative and thus the method of choice for such investigations, in particular for the morphological correlate of the three functionally defined pools of SVs.

### Comparison of TEM and FIB-SEM to investigate the neuropil and synaptic organization of the ‘hTLN’

To describe the quantitative synaptic organization of the human neocortex, biopsy tissue samples of neocortical tissue accessed during epilepsy surgery and immediately post-fixed after removal were ideally suited for high-resolution EM investigations of synaptic structures. Fine-scale TEM (Yakoubi et al. 2019a, b; Schmuhl-Giesen et al. 2022; Rollenhagen et al. 2020, 2025) or FIB-SEM (Domínguez-Álvaro et al. 2019, 2021, 2023, 2024; Rollenhagen et al. 2020; Cano-Astorga 2021; Shapson-Coe et al. 2024) are to date the methods of choice. In general, both methods can visualize key structural subelements in human tissue samples (Fig. 3).

High-confidence identification of membrane borders is a pre-requisite for the successful segmentation of synaptic key elements such as the AZs, and for elucidating the size (diameter) and distribution of SVs. At lower EM magnifications of around × 4000 to × 6000, AZs and SVs were clearly visible and distinguishable from each other in both TEM images (Figs. 3A), but also in the FIB-SEM material (Figs. 3B). However, at higher EM magnifications, fine structural elements such as the membrane areas of AZs and SVs appeared blurrier in the FIB-SEM material, so that the assessment of distance parameters became more difficult and thus non-precise (compare Figs. 3A with B, 4 with 5). The blurriness was a result of two predominant factors. First, the real point resolution of the FIB-SEM images was comparably worse in contrast to the TEM data (10,444 × 11,129-pixel Fig. 3A), and second, the signal-to-noise ratio in the FIB-SEM pictures was poorer due to the low contrast and the resulting non-optimal pixel dwell times during image acquisition. The quantitative description of the shape and size of AZs and of the pool of SVs and their distance from the PreAZ was much more difficult, so that measurements of their numbers, diameters, and distances from the PreAZ were prone to false interpretation that had an enormous impact on the definition (measurement) of AZ size and that of the three pools of SVs. This problem did not occur in the TEM processed material, where SVs could be unambiguously identified and separated from each other, even at the single vesicle resolution by the clear separation of biological membranes (compare Figs. 3A with B, 4 with 5). With that image quality, it was possible to unequivocally sort SVs into the three functionally defined vesicle pools by perimeter measurements (Fig. 2, see also chapter below, for further details see Rollenhagen et al. 2020) and TEM tomography (Fig. 7).

**Fig. 4:**
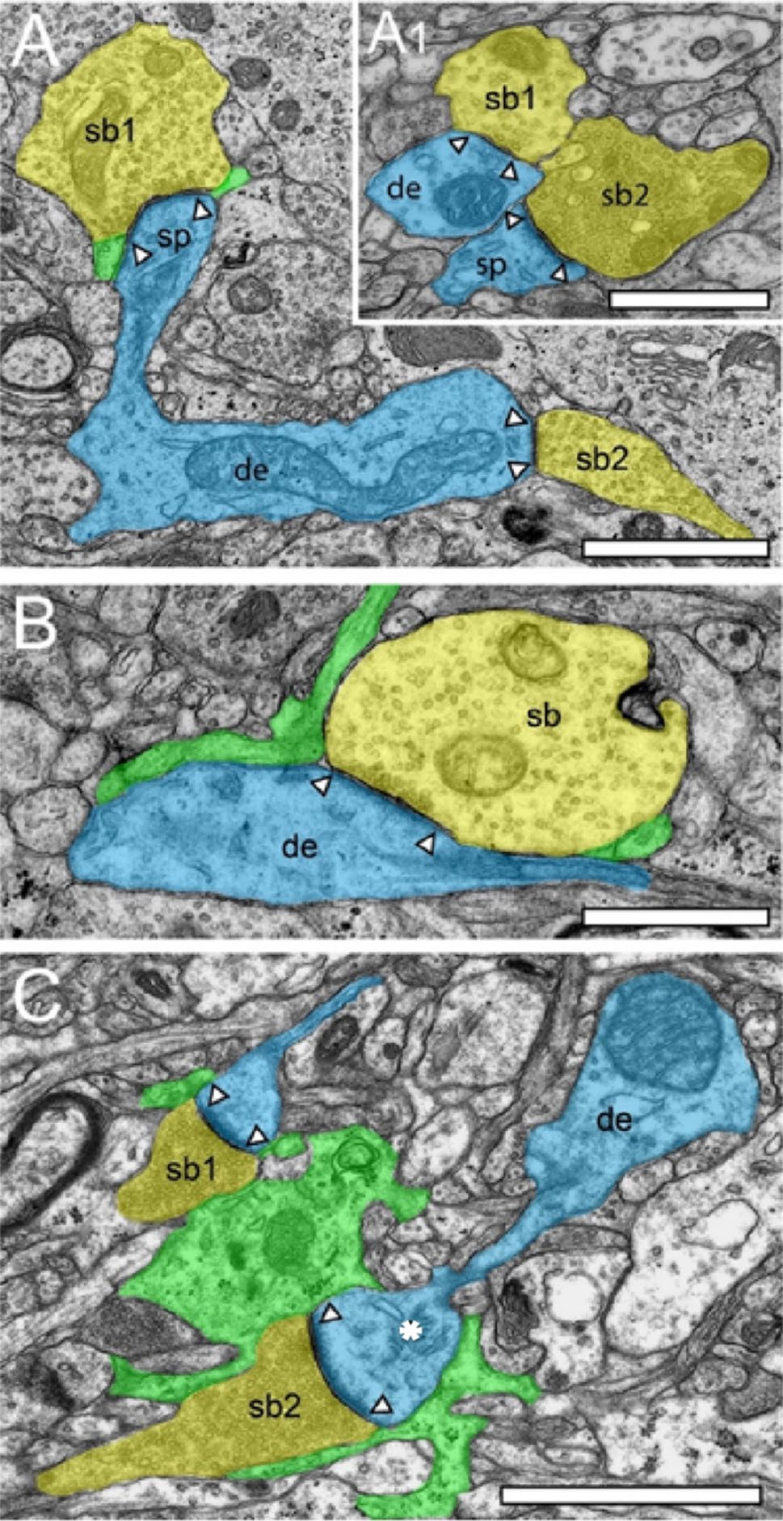
Structural characteristics of SBs and their target structures in the ‘hTLN’ as revealed by TEM. **A,** Large dendrite (de, transparent blue) with an SB terminating on a mushroom spine (sp1, transparent yellow) and the other (sb2) on a dendrite in L3 of the gyrus temporalis inferior. Scale bar 0.5 µm. **A1,** Two adjacent SBs (sb1, sb2) synapsing on a small dendrite (de, sb1) and a large spine (sp). Note that sb1 is a putative GABAergic terminal (sb) characterized by the more ovoid SVs whereas sb2 is regarded as a glutamatergic terminal. Scale bar 0.5 µm. **B,** Dendrite (de) receiving synaptic input from a large putative GABAergic SB in L1. Note the presence of fine astrocytic processes (transparent green) one of which reaches as far as the synaptic cleft. Same color code as in A. Scale bar 1 µm. **C,** Two adjacent mushroom spines (sp) emerging with a long spine neck and large spine head, one emerging from a dendrite getting input from SBs (sb1, sb2) in L3. Both synaptic complexes are tightly ensheated by fine astrocytic processes (transparent green). Same color code as in A. Note the spine apparatus (asterisk) in one of the spine heads. Scale bar 0.5 µm. In all images, AZs are marked by arrowheads

**Fig. 5:**
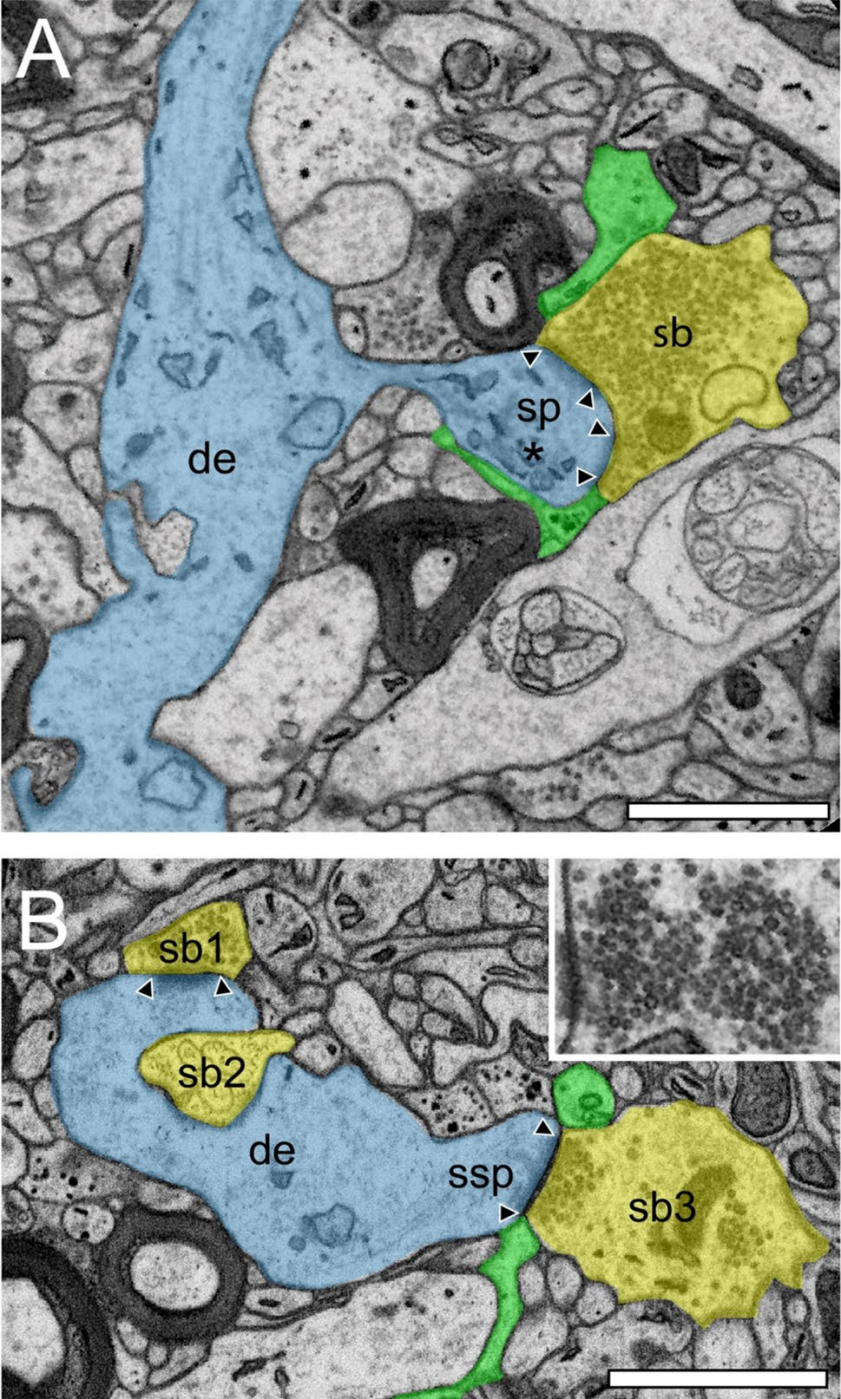
Structural characteristics of SBs and their target structures in the ‘hTLN’ as revealed by FIB-SEM. **A,** Large dendrite (de, transparent blue) with a large SB (sb, transparent yellow) with two macular, non-perforated AZs containing thousands of SVs in L3 of the gyrus temporalis inferior. Note the existence of a spine apparatus (asterisk) in the spine head. **B,** Small caliber dendrite (de) receiving input from one SB (sb1) invaginated by a second SB (sb2) and an SB (sb3) terminating on a stubby spine (ssp) in L5. Same color code as in A. Inset: pools of SVs located at a macular, non-perforated AZ. Note the blurry appearance of membranes both at the AZ and the SVs. In the two images, AZs are marked by arrowheads and astrocytic processes in transparent green. Scale bar in A and B 1 µm

Nevertheless, series of 2D images representing a stack of single-plane data of both EM methodologies could be used to generate quantitative 3D models of SBs and their target structures in the “hTLN”.

### Very special entities: SBs in the ‘hTLN’

**Fig. 6:**
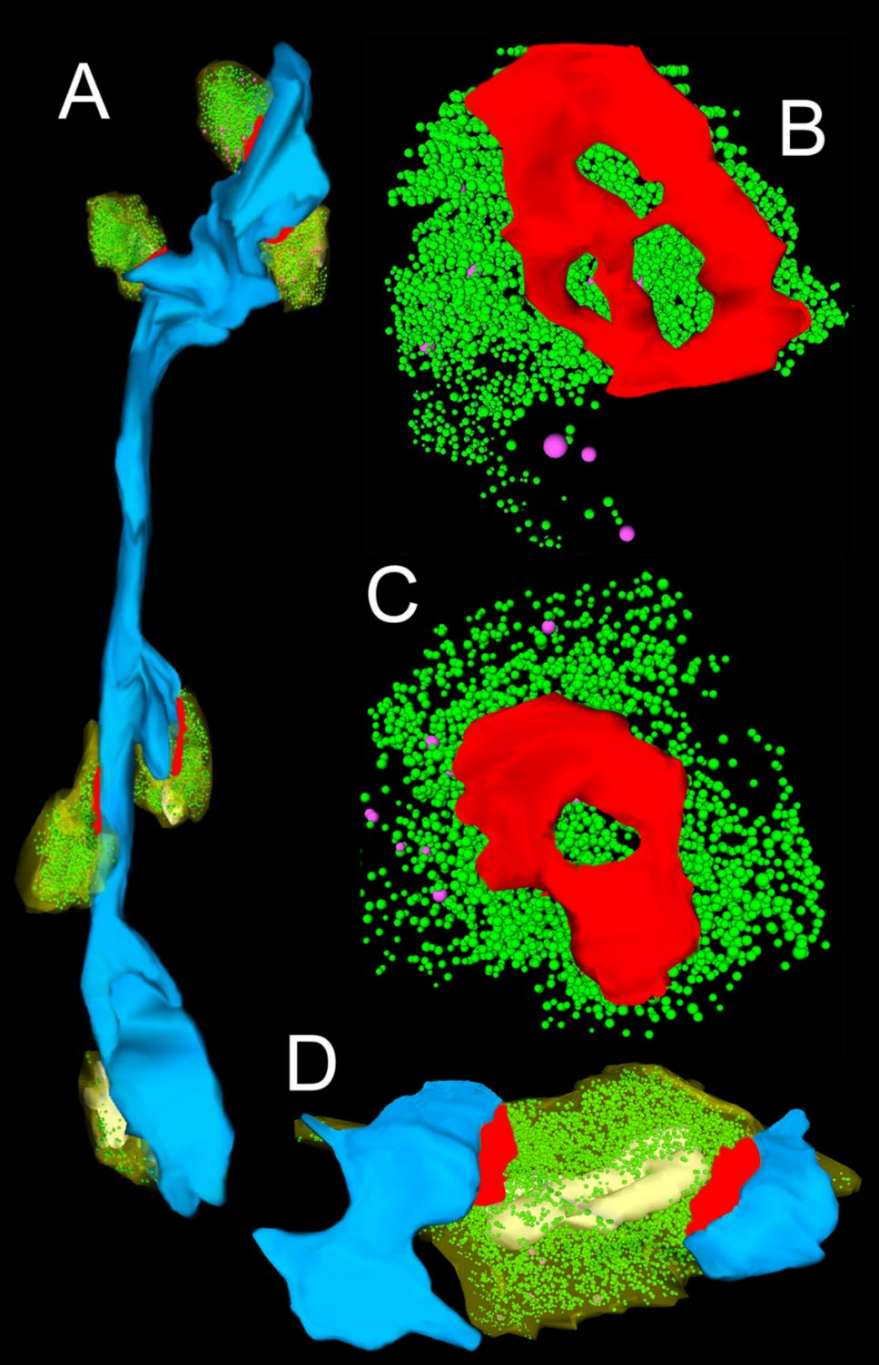
3D-volume reconstruction of synaptic structures in the ‘hTLN’. **A,** 3D-volume reconstruction of a dendritic segment in L1 (blue) with six SBs terminating on either dendrites or spines or directly on the dendritic shaft. **B, C,** Total pool of SVs (green dots) and DCVs (magenta dots) at a large AZ (red) with three perforations (B) and one with a horseshoe-like appearance (C). **D,** SB terminating on two spines. Note the mitochondria (white) associated with the pool of SVs (green dots)

**Fig. 7:**
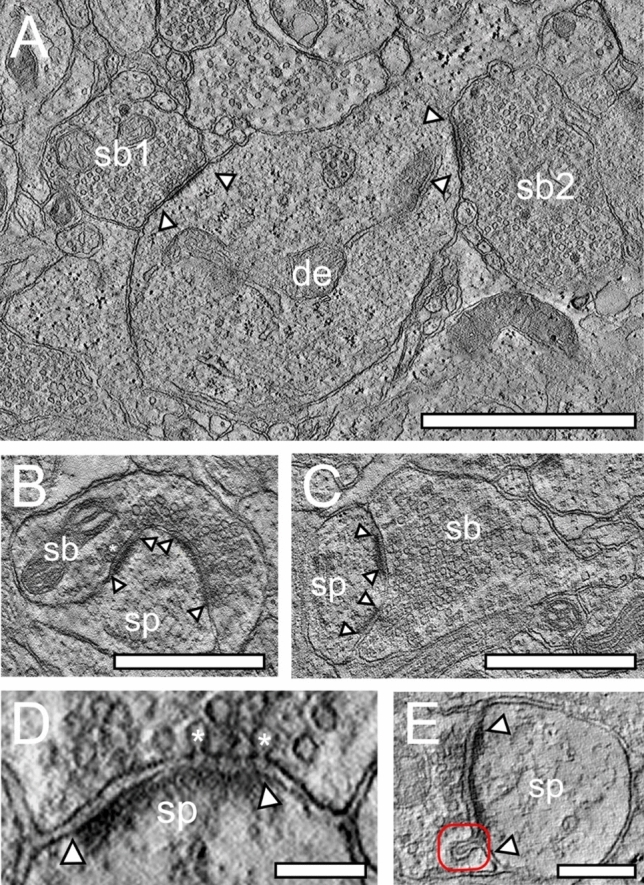
TEM tomography of synaptic complexes and ‘docked’ SVs in the ‘hTLN’. **A**, Two SBs (sb1, sb2) synapsing on a large dendrite (de) taken from a tilt-series in L4. S Scale bar 1 µm. **B, C**, Two SBs (sb) terminating on spines (sp) in L3 (B) and L5 (C). Both synaptic complexes have two macular, non-perforated AZs. Scale bar in B and C 0.5 µm. **D**, High-power single TEM image taken from a tilt-series of a spine (sp) in L1. Note the two omega-shaped bodies (asterisks) indicating the already release of neurotransmitter quanta. Scale bar 0.1 µm. **E**, High-power TEM image of a spine showing the fusing of a large DCV (framed area) at the PreAZ in L2. Scale bar 0.25 µm. In all images, AZs are marked by arrowheads

Using non-epileptic and thus non-affected neocortical access tissue taken from epilepsy surgery enabled us to study the layer-specific quantitative synaptic organization of the ‘hTLN’ (Figs. 3, 4, 5, 6, 7). So far, the following layers (L) have been quantitatively analyzed in detail: 1) L1 (Rollenhagen et al. 2025). L1 contains commissural and associational axons, inhibitory interneurons, and terminal tuft dendrites from L2, L3, and L5 pyramidal neurons. The terminal tuft dendrites in L1 are the source of Ca^2+^-spike initiation. 2) L4 (Yakoubi et al. 2019b): the receiving input layer of signals from the sensory periphery represents the first station of intracortical information processing, 3) L5 (Yakoubi et al. 2019a): the major cortico-subcortical output layer of the neocortex, and 4) L6 (Schmuhl-Giesen et al. 2022): the earliest generated cortical layer receives thalamic input. For the remaining layers L2 and L3 of the ‘hTLN’, work is in progress.

SBs in the ‘hTLN’ varied substantially in size ranging from ~ 2.5 to 15 µm^2^ with an average of ~ 5 µm^2^ throughout all layers investigated although with a skewness to smaller SBs as demonstrated by both TEM and FIB-SEM imaging. Beside similarities, striking differences in some structural parameters occurred when compared with their counterparts in experimental animals. Like in rodents and non-human primates, so-called *en passant* and end terminal SBs contacted dendritic shafts (Figs. 4A sb2, A1 sb1, 4B, 5B sb1, sb2), but the vast majority (~ 90%) of excitatory SBs were established on dendritic spines of different types including mushroom, stubby (Fig. 5B), filopodial, elongated and non-classifiable spines (Figs. 4A sb2, A1 sb2, 4C sb1, sb2, 5A). The majority of spines (~ 80–90%) contained a so-called spine apparatus (Figs. 4C, 5A), a derivative of the endoplasmic reticulum, responsible for spine motility and stabilization of the synaptic complex during single or repetitive high-frequency stimulation. Thus, it was hypothesized that spines containing this structural subelement partially contribute to the modulation of short-term synaptic plasticity (reviewed by Knott and Holtmaat 2008).

The majority of SBs in the ‘hTLN’ (~ 85%) contained a single mitochondrion or numerous mitochondria of different shape and size (Figs. 4A, B, 5A, B, 6D) occupying ~ 10–15% of the total volume of individual SBs which is comparable to numbers estimated from experimental animals. Mitochondria were always associated with SVs and transfer SVs from the resting to the RP and RRP (Verstreken et al. 2005, 2008). Thus, mitochondria in the ‘hTLN’, beside various other functions in nerve terminals, partially contribute to the re-filling and thus replenishment of the RRP and RP of SVs.

Finally, as previously shown in experimental animals, astrocytes form a dense network throughout all layers of the ‘hTLN’ (Figs. 4, 5). Synaptic complexes were either tightly ensheathed by fine astrocytic processes forming the ‘tripartite’ synaptic complex (Figs. 4A sb1, 4B, C, 5A, B sb3) separating them from adjacent synaptic complexes in the neuropil, or only a partial or none coverage (Fig. 4A sb2, A1, 5B sb1, sb2) was observed. If present, fine astrocytic processes could be followed as far as to the synaptic cleft under the pre- and postsynaptic densities (Figs. 4A sb1, 4B, C, 5A, B sb3). Two possible scenarios are most likely at AZs in the human TLN: first, astrocytes can actively take-up excessive or ‘spilled’ neurotransmitters via glutamate transporters, thereby modulating the temporal and spatial neurotransmitter concentration in the synaptic cleft and controlling the induction, maintenance, and termination of synaptic transmission. Second, only partial or no astrocytic coverage would allow the horizontal diffusion of glutamate in the synaptic cleft, thereby inducing neurotransmitter diffusion and release at neighboring AZs due to the high density of dendritic spines at pyramidal neurons, leading to glutamate spillover and synaptic crosstalk. It is most likely that both scenarios are possible at individual SBs in both experimental animals and the ‘hTLN’, although it remains elusive which scenario is more efficient.

The most striking difference between SBs in the ‘hTLN’ when compared to non-human primates and rodent neocortex is the shape and size of the AZs and that of the three functionally defined pools of SVs, namely, the RRP, RP, and resting pool. Although comparably small in average size (see above), excitatory SBs in L1 to L6 of the human ‘hTLN’ contained AZs that were on average two-to-threefold larger in size (~ 0.2–0.3 µm^2^ in surface area) when compared to SBs of the equivalent size category in rodents or non-human primates. This applies to even much larger CNS synapses such as the cerebellar climbing fiber (~ 0.14 µm^2^) and mossy fiber boutons (~ 0.13 µm^2^), hippocampal mossy fiber boutons (~ 0.17 µm^2^) terminating on spiny excrescences of apical dendrites of CA3 pyramidal neurons and the Calyx of Held-principal neuron synapse in the medial nucleus of the trapezoid body (~ 0.1 µm^2^). In numerous (50–60%) SBs of the ‘hTLN’, the AZs covered most of the pre- and postsynaptic apposition zone at spines (Figs. 4, 5) hence enlarging the presynaptic ‘docking’ zone for SVs. In addition, some of the SBs contained not only a single but up to three AZs (Fig. 6D) in the ‘hTLN’.

In addition to macular, non-perforated AZs (Figs. 4A-C, 5B sb1, sb3), we also observed perforated, ring- and horseshoe-like AZs (Figs. 5A, 7B, C) with a skewness to macular AZs. The size of the synaptic cleft was on average ~ 20–25 nm with slight differences between layers, but comparable with findings in experimental animals.

Even more strikingly, SBs in the ‘hTLN’ contained a total pool of SVs (average 1100—3000 SVs) that was in general three-to-sixfold larger than that reported in the rodent and non-human primate neocortex; however, quite variable in individual SBs (minimum 250; maximum 15,000) like in experimental animals and showed layer-specific differences. These high numbers in the total pool suggested also comparably large RRPs, RPs, and resting pools. Indeed, using a perimeter analysis, the putative RRPs were on average by three-to-fivefold, the putative RPs by ~ two-to-threefold and the putative resting pools on average by twofold larger than in rodent and non-human primate neocortex. Using a perimeter (p) of 20 nm criterion which is less than a single vesicles diameter, the number of SVs in the putative RRP further increased by up to fourfold when compared to experimental animals but showed layer-specific differences. It has been shown recently that the size of the RRP dynamically regulates multi-vesicular release in mice (Vaden et al. 2019) and this seemed to be also the case at SBs in the ‘hTLN’.

Thus, these large pools suggest reliable synaptic efficacy and strength even at high-frequency stimulation; hence, a rapid depletion of the RRP and RP could be prevented by replenishment of SVs from a large resting pool. It has to be noted though that a huge variability exists in the structural composition of individual SBs in relation to layers in the ‘hTLN’. In consequence, the variable size of the putative RRP, the putative RP, and the putative resting pool may partially contribute to the modulation of synaptic plasticity in a layer-specific manner.

Taken together, the structural composition of both the presynaptic terminal and the postsynaptic spine as the main target structure, together with the tight ensheathment of fine astrocytic processes, suggests high synaptic efficacy and reliability of synaptic transmission but also in the induction, regulation, and termination of short-term plasticity at SBs in the human ‘hTLN’.

### Comparison of TEM and FIB-SEM to investigate the neuropil and synaptic organization of the ‘hTLN’

It is still controversially discussed whether TEM or FIB-SEM represents the best-suited imaging modality to characterize the organization of the neuropil and its components. In summary, both methods are suitable to visualize key ultrastructural subelements, but with our so far established and refined sample preparation and image acquisition protocols, the contrast in the resulting FIB-SEM images was clearly poorer compared to corresponding TEM images. This was, in particular, critical for the visualization of biological membranes (compare Fig. 3A [TEM] with Fig. 3B [FIB-SEM], Fig. 4 [TEM], and Fig. 5 [FIB-SEM]). The doubtful identification of the membrane borders was a pre-requisite for the successful segmentation of synaptic key elements, such as the AZs and for elucidating the size and shape of SVs. At lower magnifications of around × 6000 to × 8000, the shapes of AZs and SVs were clearly visible and distinguishable from each other in the TEM images (Figs. 3A, 4), but also visible in the FIB-SEM images (Figs. 3B, 5). However, at higher magnifications, fine structural elements such as membrane areas of AZs and SVs appeared more blurry in the FIB-SEM material, so that the assessment of distance parameters became more difficult and thus non-precise. The blurriness was a result of two predominant factors. First, the real point resolution of the FIB-SEM images was worse in contrast to the TEM data (10,444 × 11,129 pixel; Fig. 3A), and second, the signal-to-noise ratio in the FIB-SEM pictures was poorer due to the low contrast and the resulting non-optimal pixel dwell times during image acquisition. The quantitative description of the SVs and their AZ environment was very difficult, so that measurements of their numbers, diameters, and distances from the presynaptic density were prone to false interpretation, which had an enormous impact on the definition (measurement) of the RRP and RP. This problem did not occur in the TEM processed material, where SVs could be unequivocally identified and separated from each other, even at the single vesicle resolution. With that image quality, it was possible to sort SVs into the three functionally defined vesicle pools by perimeter measurements.

### TEM tomography in the ‘hTLN’

Technological advances in TEM tomography and Cryo-EM have opened a new door to image directly the sub-cellular and molecular organization of the pre- and postsynaptic density and have promised new conceptual breakthroughs in the future. For example, the molecular organization of the AZ and the pool of SVs, particularly that of the RRP, demonstrated a remarkable structural heterogeneity at the PreAZ between individual SBs but also in different brain regions and areas. This allows correlating structural heterogeneity with the functional characteristics of individual synapses as revealed by TEM tomography.

However, to our knowledge, TEM tomography had never been performed on human tissue samples (Fig. 7). Hence, we have used this approach to directly compare our perimeter measurements of the putative RRP (see above) by counting the number of so-called ‘docked’ SVs or already fused omega-shaped SVs (Fig. 7D). The majority of PreAZs (~ 98%) in all layers of the ‘hTLN’ SBs contained more than 2 ‘docked’ SVs.

Both TEM tomography and perimeter measurements reveal layer-specific differences, with both values in the RRP ranging between 2 and 8 SVs (Yakoubi et al. 2019a, b; Schmuhl-Giesen et al. 2022; Rollenhagen et al. 2025). It has to be noted that the number of SVs in the RRP and RP was in general higher in all layers of the ‘hTLN’ when compared with findings in the adult rat somatosensory cortex (Rollenhagen et al. 2015, 2018). Strikingly, no or only a weak correlation between the size of SBs, the size of the presynaptic density, and the RRP and RP was found in both experimental animals and in the ‘hTLN’. These findings point to an independent regulation of the RRP and RP at SBs in the ‘hTLN’. In summary, TEM tomography verified and supported our perimeter analysis and appeared to be well suited for the estimation of vesicle pools, most importantly for that of the RRP in the ‘hTLN’.

## Discussion

Here, we demonstrated that TEM, FIB-SEM, and TEM tomography are well suited to quantitatively describe the synaptic organization of the ‘hTLN’. This approach allowed the investigation of structural and synaptic parameters representing morphological correlates of synaptic transmission and plasticity that can be used to correlate structure with function. In addition, a direct comparison of synaptic structures obtained in humans with findings in experimental animals becomes more possible. Furthermore, the generation of quantitative 3D models of SBs and their target structures could be used for numerical and/or MonteCarlo simulations of various synaptic parameters, still only partially assessable for experiments, at least in humans.

### Methodological considerations

#### ‘Post-mortem’ vs. biopsy tissue samples

For this study, neocortical brain tissue was used from patients that underwent epilepsy surgery due to drug-resistant temporal lobe epilepsy. Previous studies confirmed that such tissue samples taken from epilepsy or tumor surgery are best suited for structural TEM and FIB-SEM investigations on the cellular, sub-cellular, and synaptic level and functional in vitro studies (Kirkpatrick et al. 2006; DeFelipe et al. 1999; Alonso-Nanclares et al. 2008; DeFelipe 2011; Blazquez-Llorca et al. 2013; Bernhardt et al. 2013; Kay et al. 2013; Liu and Schumann 2014; Testa-Silva et al. 2010, 2014; Mohan et al. 2015; Molnár et al. 2016; Seeman et al. 2018a, b; Yakoubi et al. 2019a, b; Schmuhl-Giesen et al. 2022; Cano-Astorga et al. 2021, 2023, 2024; Rollenhagen et al. 2025). Prior to surgery, all patients underwent high-resolution MRI, multielectrode electrophysiology and long-term video EEG monitoring. In our cases, since the epileptic focus was found exclusively in the hippocampus without affecting the neocortex, tissue samples can be regarded as non-affected. However, one major drawback of using biopsy tissue samples is the availability of suitable tissue samples from epilepsy or tumor surgery due to strong ethical regulations and the comparatively small number of patients.

So-called ‘post-mortem’ donor brains are used worldwide for other investigations, for example for mapping and parcellating the human brain (von Economo and Koskinas 1925; Betzel et al. 2022; reviewed by Buckner and DiNicola 2019), or receptor autoradiography (Caspers et al. 2013; Murgaš et al. 2022; Rapan et al. 2022) or intracellular Lucifer yellow injections (Buhl and Lübke 1989; Benavides-Piccione et al. 2024; Turegano-Lopez et al. 2024). However, ‘post-mortem’ brains are unfortunately only partially suited for investigations of synaptic structures. The advantage of using such brains is their greater availability when compared with biopsy tissue samples taken during surgery. The major drawback using ‘post-mortem’ brains is the time-window between the decease of the donor and the removal of the brain. Long delays of about 1 day before removal cause severe ultrastructural changes within the tissue from decomposing and hypoxia-mediated autolysis of biological membranes immediately occurring after death and before fixation. Such changes partially distort critical structural features, including for example, distortions of pre- and postsynaptic densities and membranes of SVs rendering such samples only partially suitable for creating quantitative 3D synaptic models. However, if the time between the decease of the patient and the removal of the brain falls within a time-window of 3–4 h after death and the brain is fixed immediately after removal, these brains can be considered adequate for detailed quantitative ultrastructural analysis (see for example Cano-Astorga et al. 2021, 2023, 2024). In general, working with human tissue samples is still a challenging task due to strict ethical regulations.

#### TEM vs. FIB-SEM examination and features

Ways to investigate the synaptic organization of the brain in such great detail are either based on serial ultrathin sections using TEM imaging or *block face* imaging using FIB-SEM. While for TEM, a selected region of interest (ROI) within a series of consecutive ultrathin sections is examined and documented by digital images, such image stacks are generated inside the FIB-SEM by constant defined layer milling of the ROI using a focused gallium ion beam in combination with an automated image acquisition right after every removed z-layer. From the resulting z-stacks of EM images using *both* EM techniques, quantitative 3D models of SBs and their prospective target structures could then be generated using different commercially or self-made reconstruction software tools running on high-performance computer systems (Alonso-Nanclares et al. 2008; Blazquez-Llorca et al. 2013; Yakoubi et al. 2019a, b; Schmuhl-Giesen et al. 2022; Cano-Astorga et al. 2021, 2023, 2024; Rollenhagen et al. 2020, 2025; Shapson-Coe et al. 2024).

It should be mentioned first that both imaging techniques, TEM as well as FIB-SEM, have advantages, but also disadvantages (see below). Serial sectioning and subsequent TEM examination are a very labor-intensive and thus time-consuming process with a comparably low throughput of tissue samples. Table 1 summarizes the total time of all individual tissue processing steps required to generate an aligned image z-stack compared for TEM or FIB-SEM.

**Table 1:** Summarizes all required steps from sample preparation, data acquisition and 3D-volume reconstructions for TEM and FIB-SEM

Experimental procedere	Time	
	TEM	FIB-SEM
Sample preparation (fixation, vibratome sectioning, osmification, dehydration, embedding, polymerization	120 h	125 h
Sputter coating, sample transfer into SEM + sample stabilization, deposition of protective metal pad, trench milling and polishing of acquisition plane	not applicable	5 h
Data acquisition	1–4 weeks*	24—30 h**
Data post-processing (parameter adjustment, bright/contrast, stack alignment, potential cropping)	not applicable	1 h
Total time	~ 245 – 485 h ≅ 10–20 days	~ 160 h ≅ 7 days

^*^data acquisition critically depends on the number of ultrathin sections/series and regions of interest

^**^data acquisition time critically depends on the size of the z-stack and size of the ROI

Second, in ultrathin sections, the tilting of the electron beam restricts the ROI to a maximal area of ~ 16.5 × 16.5 µm and during the cutting and imaging process, malformations, or distortions or even the loss of some ultrathin sections within the series can be a limiting factor. In addition, since serial sections are placed on Formvar-coated slot copper grids instead of line grids, tracking corresponding structures in the selected ROI could lead to an extreme burden and finally to burning of the film using high voltage. However, the major advantage using serial ultrathin sections and subsequent TEM imaging is their very high quality, reaching individual vesicle resolution. This is a pivotal requirement for the detailed analysis of important structural subelements, such as the number, size and shape of AZs, and the organization and size of the three functionally defined pools of SVs that allow the generation of quantitative 3D models of synaptic structures.

In contrast, FIB-SEM, a relatively new, modern TEM technology, allows a much higher throughput of tissue samples, because the time and labor-intensive step of serial ultrathin sectioning is no longer required. Second, a larger area of interest ~ 22.97 × 19.81 µm or even larger areas depending on the required resolution can be obtained compared to TEM. Finally, since the surface of the intact block is milled and polished, no malformations or distortions of individual images are expected, so that only minor alignment processes of adjacent EM images are required in the image post-processing phase. Besides these two major advantages of FIB-SEM, the speed in data acquisition and the possibility to investigate larger ROIs and nearly unlimited z-dimension in an individual analysis, this technique is still rather challenging with respect to optimizing the resolution of images. Concerning image quality, the first critical factor is the resolution potential of the underlying imaging system, FIB-SEM vs. TEM. Regarding this parameter, one has to consider that an SEM has indeed a poorer resolution than a TEM of comparable technical standard. This could be mainly attributed to the standard sample thickness and the bulb-shaped electron beam interactions in deeper areas of the tissue sample. Modern FIB-SEM has a maximum point resolution of about 1 nm under optimal conditions, whereas a comparable TEM reaches resolution limits of about 0.1 nm. In practice, with the relatively thick resin-embedded samples, the best final resolution that could be obtained with FIB-SEM was between 3 and 5 nm in x–y dimension. Hence, the practical resolution of an FIB-SEM at present is not sufficient for a detailed study of vesicle fusion events at AZs. Even for the quantification and classification of SVs with a size of about 30–40 nm, a resolution of 5 nm should indeed be sufficient to identify and discriminate SVs. However, due to a poorer resolution at appropriate higher magnifications, it is hard to separate individual SVs from each other, which may result in either an over- or underestimation.

Nevertheless, our examples of AZ and SV visualization by FIB-SEM delivered a slightly poorer image result compared to the TEM data. At higher magnification, FIB-SEM images appeared significantly blurrier compared to corresponding TEM pictures, and as a result, it became harder to discriminate between individual SVs with a consequent negative effect on all subsequent quantification and classification approaches. Using well-studied standard TEM sample preparation protocols, it is relatively easy to acquire TEM images with optimal contrast and good signal-to-noise ratio. Along the workflow for an FIB-SEM analysis, there are many more parameters that must be considered to optimize the image result. These options start with the general sample preparation procedure. Using SEM, the likelihood that scattering effects leading to secondary and back-scattered electrons (leaving the sample in an upwards direction to contribute to the image formation) is quite low, so that the overall contrast of an SEM image is generally poorer compared to a TEM picture. As a consequence, the respective FIB-SEM users are in constant search for improved sample preparation protocols through which more electron-dense molecules get deposited at defined ultrastructural elements and thereby increase the structural contrast (see for example Deerinck et al. 2010). Ideas for optimizing the sample preparation include the use of conductive elements inside the embedding resin to increase the discharge capacity of the specimen by further improving charging and resolution of acquired images in the electron microscopic volume imaging (Nguyen et al. 2016). This in turn would reduce charging effects, which contribute to sample instability during image acquisition.

For the same reason, one has to optimize the thickness and composition of the sputter-coat, and it could be meaningful to also address the overall sample thickness which might have a negative effect on the thermic stability of the sample.

Apart from the blurry visualization of the target structures at high magnifications using FIB-SEM, they also appeared structurally different from the TEM images. Potentially, this was due to the use of two individual buffer systems for the different sample preparation protocols (PB vs. CB).

In summary, there is still an abundance of parameters in the context of FIB-SEM sample preparation and image acquisition, which can be optimized to potentially fulfill needs for a proper and reliable quantification and classification of the SV and AZ compartments in human brain biopsies. In combination with the much faster data acquisition speed and the option to analyze larger sample volumes, FIB-SEM would then be a real alternative to TEM.

In future, the combination of both TEM and FIB-SEM and further developments in fixation and embedding protocols will represent the comprehensive toolbox which will be inevitably needed to address specific questions regarding the quantitative geometry of synaptic complexes and to further unravel the synaptic ‘microcosm’ of the brain. In this context, the description of ‘connectomes’ and the synaptic organization of various layers, nuclei and regions of the human brain will be of particular interest (see for example Middlebrooks and Grewal 2022; Shapson-Coe et al. 2024; reviewed by Bazinet et al. 2023; Seguin et al. 2023a, b).

#### Counting SV populations

Knowledge about the total pool of SVs and that of the three functionally defined pools namely the RRP, RP, and resting pool would allow concrete statements about the ‘behavior’ of a given SB. In particular, the number of SVs in the RRP and RP, but also the resting pool and their replenishment and re-filling rates at a given SB are crucial parameters that govern und underlie synaptic transmission and plasticity. Thus, electron microscopy is an important technique for the study of synaptic morphology and its relation to synaptic function. The data analysis for this task requires the segmentation of the relevant synaptic structures, such as synaptic vesicles, active zones, mitochondria, presynaptic densities, synaptic ribbons, and synaptic compartments. Counting SVs is a time-consuming and labor-intensive job that could last, depending on the size of individual SBs, several weeks. Thus, nowadays, several attempts have been made to count SVs automatically using self-developed algorithm-driven software packages. So far, this has been performed either on digital EM images of SBs (Imbrosci et al. 2022; Muth et al. 2024) or individual tilt-series using EM tomography (Muth et al. 2024). First results are quite promising in counting structural and synaptic parameters but this has to be further developed for a much broader use.

#### Perspective to work with human tissue samples

Numerous studies have shown that neocortical access tissue can be considered ‘normal’, unaffected tissue if the tissue samples were taken far away from the epileptic focus or tumor and monitored by electroencephalography, direct electrophysiology, and magnetic resonance imaging before surgery. These investigations provide the basis to compare ‘healthy’ human brain tissue with that of seizure (epileptic-) or tumor-affected tissue. We recently started to investigate ‘epileptic’ neocortical tissue samples from drug-resistant patients that had not undergone epilepsy surgery for years. Preliminary results demonstrated that depending on the number of years since surgical intervention, the affected neocortex developed dramatic signs of degeneration in the neuronal and synaptic organization of the neuropil (Lübke and co-workers, personal observations). Using the same experimental approach as described above, we started to investigate ‘affected’ neocortical tissue samples to look whether the arrangement of synaptic complexes, synaptic targeting, or the quantitative morphology of SBs will undergo dramatic changes and how that would influence their function. This can be further achieved by realistic numerical and/or MonteCarlo simulation based on quantitative 3D models of synaptic structures of various parameters of the signal cascades, for example Ca^2+^ dynamics, transmitter release, and diffusion via the synaptic cleft on the presynaptic site and receptor sensitization and de-sensitization postsynaptic, that underlie synaptic transmission and plasticity.

Much more and extensive work is needed to unravel the synaptic and molecular organization of the normal and pathologically altered human brain.

## Data Availability

The data that support the findings of this study are available upon reasonable request from the corresponding author.
